# Correlation between Sirtuin 1 downregulation and reduced vitamin D receptor expression in patients with diabetic neuropathy

**DOI:** 10.1007/s00592-025-02463-w

**Published:** 2025-02-20

**Authors:** Andrea Latini, Giada De Benedittis, Chiara Morgante, Beatrice Gasperini, Ilenia D’Ippolito, Davide Lauro, Giuseppe Novelli, Cinzia Ciccacci, Vincenza Spallone, Paola Borgiani

**Affiliations:** 1 Department of Biomedicine and Prevention, Genetics Section, University of Rome Tor Vergata, 00133 Rome, Italy; 2 Department of Systems Medicine, Endocrinology Section, University of Rome Tor Vergata, 00133 Rome, Italy; 3 Department of Pharmacology, School of Medicine, Reno University of Nevada, Reno, NV 89557 USA; 4 UniCamillus, Saint Camillus International University of Health Sciences, 00131 Rome, Italy

**Keywords:** Diabetic neuropathy, SIRT1, Vitamin D receptor

## Abstract

**Aims:**

We aimed to analyse Sirtuin 1 (SIRT1) and Vitamin D receptor (VDR) expression levels in the peripheral blood of patients with type 2 diabetes (T2D), characterized for the presence of diabetic neuropathy (DN), and to evaluate possible genetic factors that could influence the VDR expression levels.

**Methods:**

Fifty-one participants with T2D, who underwent neurological assessment for DN were recruited. We quantified the mRNA levels of SIRT1 and VDR in peripheral blood mononuclear cells. Moreover, we analysed the methylation status and the rs2228570 genetic variant of VDR promoter.

**Results:**

Patients with DN (*n* = 32) showed lower expression of SIRT1 (p_corr_=0.018) and VDR (p_corr_=0.009), compared to those without DN. Furthermore, we observed a positive correlation between the mRNA levels of SIRT1 and VDR (*p* = 0.01). The expression levels of these genes negatively correlated with the score based on cardiovascular reflex tests (CARTs score). Moreover, the variant allele of rs2228570 in the VDR gene was associated with higher expression of this gene compared to the wild-type allele (*p* = 0.003).

**Conclusion:**

In patients with DN, both SIRT1 and VDR expression levels are reduced and interrelated. Low VDR expression levels could negatively affect SIRT1 transcription, thus influencing all the most pathogenetic pathways of DN regulated by this protein.

## Introduction

Diabetic neuropathy (DN) is one of the most prevalent and disabling complication of type 2 diabetes (T2D). The main forms of DN, diabetic polyneuropathy (DPN) and cardiovascular autonomic neuropathy (CAN), approximately affect 30% and 20% of patients with diabetes, respectively, but their frequency significantly increases with age and a longer history of diabetes, until more than 50% [1, 2]. DPN increases the risk of foot ulceration through the loss of protective sensation, and may cause neuropathic pain with heavy impact on quality of life. Moreover, DPN and CAN are associated with a twofold and threefold increase in the risk of mortality, respectively [3, 4]. All these aspects highlight the need for effective prevention and treatment strategy.

Although the pathogenesis of DN is not yet fully understood, various factors and underlying mechanisms have been identified, like hyperglycemia and dyslipidemia with the downstream effects of formation of advanced glycation end products (AGEs), oxidative stress, endothelial dysfunction, endoplasmic reticulum stress, mitochondrial dysfunction, inflammatory responses, leading to damage and neuronal cell death [5, 6].

Sirtuins, known also as nicotinamide adenine dinucleotide-dependent (NAD+-dependent) deacetylases, are a family of class III histone deacetylases involved in a number of important biological processes, including metabolism, cellular longevity and stress response [7]. Sirtuin1 (SIRT1) is considered a metabolic sensor since it responds to changes in energy status and regulates mitochondrial function, oxidative stress, inflammation and cell death, all phenomena involved in DN [8]. For these reasons, several preclinical studies suggested that activation of SIRT1 could attenuate DN symptoms. For instance, in vivo studies in mice have shown that overexpression of SIRT1 in neurons prevents and reverses DN [9] and alleviates neuropathic pain by regulating the synaptic plasticity [10]. Other studies indicated that the upregulation of SIRT1 expression in sciatic nerve tissue improved nerve conduction velocity [11]. Despite the evidence emerging from in vitro and in *vivo* experiments, studies on SIRT1 levels in patients with DN have never been conducted. Furthermore, it is not clear which kind of mechanism involved in DN could affect the production of SIRT1.

In the promoter region of the SIRT1 gene maps a vitamin D response element (VDRE), a DNA sequence to which the vitamin D receptor (VDR) binds when complexed with the active form of vitamin D. Therefore, vitamin D promotes SIRT1 expression by direct interaction between VDR and SIRT1’s promoter [12]. Moreover, VDR can induce SIRT1 expression in a ligand-independent manner, through the interaction with the transcription factors FOXOs [13]. We decided to analyze the SIRT1 and VDR expression levels in peripheral blood of patients with T2D who underwent neurological evaluation for DPN and CAN. We then explored the methylation status and a functional single nucleotide variant of VDR promoter, to evaluate a possible association between these factors and VDR expression levels.

## Materials and methods

###  Patients’ recruitment and sample collection

A total of 51 T2D patients, who underwent neurological evaluation for DPN and CAN, were recruited from the diabetic clinic of the Tor Vergata University Hospital in Rome (Italy).

The inclusion criteria were a diagnosis of T2D and age between 18 and 80 years. The exclusion criteria included the presence of neuropathy due to other causes than diabetes, conditions potentially responsible for autonomic dysfunction, severe comorbidities (such as malignancies, recent cardiovascular events, heart failure, advanced renal failure or liver disease), advanced peripheral arterial disease, severe psychiatric disorders or any other condition that prevents the understanding of the questionnaires.

Complete clinical history was recorded regarding diabetes, comorbidity, and any potential cause of polyneuropathy. Clinical parameters and neurological evaluation were described in a previous paper Ciccacci et al. [14]. Briefly, DPN diagnosis was based on the presence of two abnormalities among neuropathic symptoms, signs (assessed using Michigan Neuropathy Screening Instrument questionnaire and Michigan Diabetic Neuropathy Score) [15], vibration and thermal perception thresholds [16]. CAN diagnosis was based on at least one abnormality among four cardiovascular reflex tests (CARTs), i.e., heart rate response to deep breathing, lying to standing, and Valsalva manoeuvre and orthostatic hypotension test [17].

The study was approved by Ethics Committee of the University Hospital of Rome Tor Vergata (Approval No. 2936/2017). Informed written consent was obtained from each patient.

###  RNA extraction and qRT-PCR analysis

Total RNA was isolated from peripheral blood mononuclear cells (PBMCs) using the TRIzol reagent (Ambion, CA, USA) protocol, followed by reverse transcription using the High Capacity cDNA Reverse Transcription Kit (Applied Biosystems, Waltham, MA, USA). *SIRT1* and *VDR* expression analysis was performed by quantitative RT-polymerase chain reaction (SYBR Green Assay, Applied Biosystems) using the 7500 Real-Time PCR System (Applied Biosystems, Foster City, CA, USA). Each expression analysis was performed in triplicate. The relative difference in *SIRT1* and *VDR* gene expression levels was calculated using the 2^−ΔΔCt^ method normalized to an endogenous control (β-Actin). Data have been reported as mean values ± standard deviation.

### DNA extraction and genotyping

Genomic DNA was isolated from PBMCs using a Qiagen blood DNA mini kit and genotyping analyses have been performed by allelic discrimination assays with TaqMan technology (Applied Biosystems, Foster City, CA, USA). Fok1 polymorphism (rs2228570) in the promoter region of *VDR* gene was investigated. In each run, samples with known genotypes, previously identified by direct sequencing, have been included.

### Methylation analysis

A pyrosequencing analysis was conducted in order to investigate a possible correlation between methylation signature and *VDR* expression levels. Six CpGs within a 104-bp region located in the VDR gene promoter region were analysed. An amount of 400 ng of DNA was used for bisulfite conversion performed by the EZ DNA Methylation-Gold kit (Zymo Research, Irvine, CA, USA), following the manufacturer’s instructions. After bisulphite conversion, the genomic DNA was quantified by DS-11 Spectrophotometer (DeNovix). A total of 10 ng of each converted DNA was amplified with the PyroMark PCR kit (Qiagen, Hilden, Germany). All products were sequenced using PyroMark Gold Q24 reagents (Qiagen, Hilden, Germany) in combination with the PyroMark Q24 platform (Qiagen, Hilden, Germany) according to the manufacturer’s instructions. The pyrogram traces generated with distinct peaks were subsequently analyzed and the methylation levels at different CpGs were calculated by the PyroMark Q24 software, version 2.0.7 (Qiagen, Germany).

###  Statistical analysis

SPSS Programme v.26 (IBM Corp, Armonk, NY, USA) was used for all statistical analyses and all graphs were performed by GraphPad Prism 9 (GraphPad Software, USA). The expression levels of each sample have been analysed in triplicate and data were reported as mean values ± standard deviation. The analysis of variance (ANOVA) test has been used to compare gene expression and methylation values among the different phenotypic and genotypic groups. Pearson correlation analyses was used to evaluate a possible linear relationship among the expression of *SIRT1* and *VDR* genes and the neurological score of T2D patients, for each subgroup of DN. For all analyses, significance was set at p-value ≤ 0.05.

## Results

We included 51 T2D participants (34 men), with a mean age of 62.51 ± 6.68 years, a diabetes duration of 12.27 ± 9.21 years, body mass index (BMI) of 31.51 ± 6.23 kg/m2, and HbA1c of 7.14 ± 1.47 (Table 1). Vitamin D supplementation due to deficiency was present in 22.4% of subjects. Among participants, after neurological assessment, 32 (62.7%) satisfied the diagnostic criteria for DN (29 for DPN and 22 for early and confirmed CAN).

**Table 1:** Clinical and anthropometric characteristics of the 51 T2D patients.

Females/males	17/34
Age (years)	62.5 ± 6.7
Disease duration (years)	12.3 ± 9.2
BMI (kg/m^2^)	31.5 ± 6.2
Insulin treated (%)	19.6
HbA1c (%)	7.3 ± 1.5
HbA1c (mmol/mol)	54.5 ± 16.2
Total cholesterol (mg/dl)	170.6 ± 37.1
HDL cholesterol (mg/dl)	46.7 ± 13.6
Triglycerides (mg/dl)	145.0 ± 131.5
eGFR (ml/min)	94.0 ± 34.5
With microalbuminuria (%)	19.6
With dyslipidemia (%)	92.2
Casual systolic blood pressure (mmHg)	138.2 ± 15.5
Casual diastolic blood pressure (mmHg)	81.1 ± 17.0
With hypertension (%)	80.4
With peripheral arterial disease (%)	9.8
With diabetic retinopathy (%)	23.5
With cardiovascular disease (%)	15.7
Current smokers (%)	58.8
Regular physical activity (%)	66.7
Alcohol consumption (%)	33.3
Patients with Diabetic Neuropathy (%)	62.7
Patients with DPN (%)	56.9
Patients with CAN (%)	43.1
Vitamin D Supplementation (%)	21.3

**Fig. 1:**
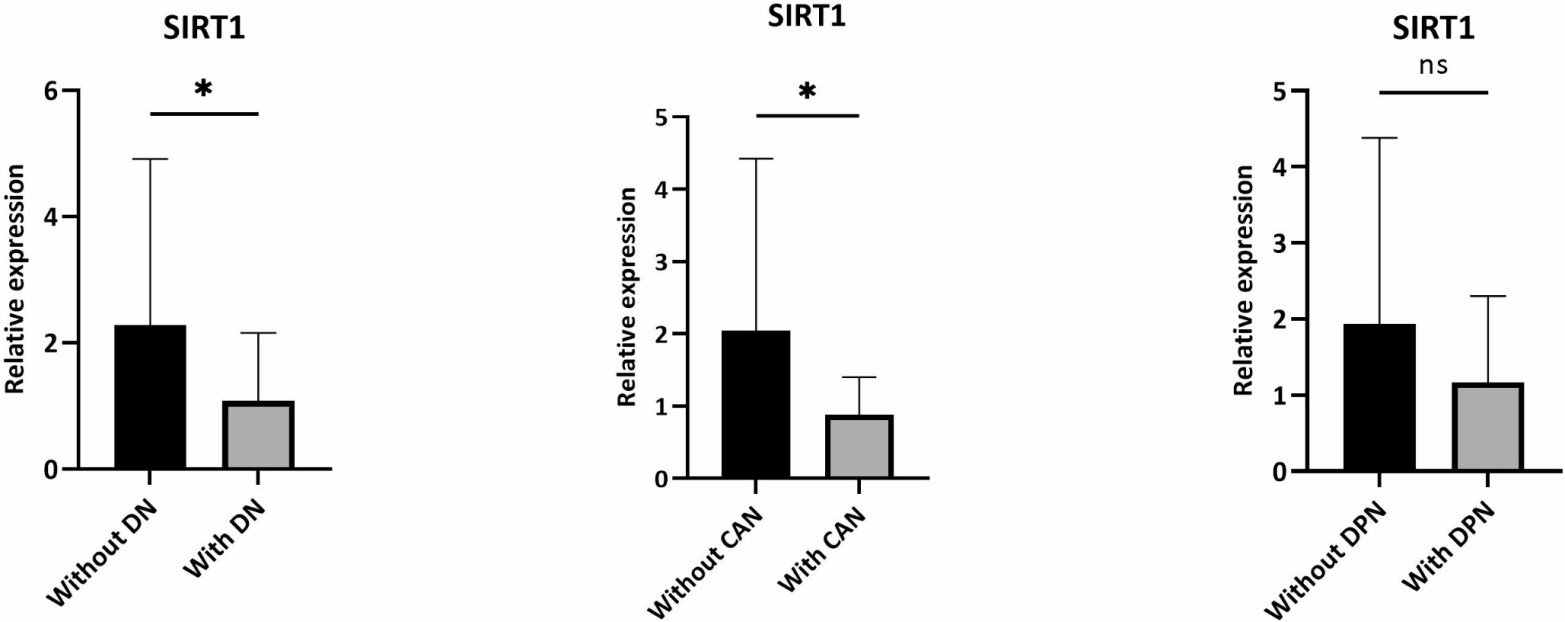
Comparisons of SIRT1 expression levels in different subgroups of diabetic patients. * = p-value < 0.05. DN: Diabetic neuropathy; CAN: Cardiovascular autonomic neuropathy DPN: Diabetic polyneuropathy.

As shown in Fig. 1, patients with DN show low levels of SIRT1 compared with those of patients without DN (*p* = 0.039). The statistical significance was confirmed and improved after correction by age, sex, diabetes duration, BMI and HbA1c (p_corr_ = 0.018). In particular, stratifying subjects for the specific form of neuropathy, we observed that patients with CAN showed a significant decrease in SIRT1 expression levels (*p* = 0.037), also after correction by age, sex, duration, BMI and HbA1c (p_corr_ = 0.022). As shown in Fig. 1, we observed a decrease in SIRT1 expression levels even in subjects with DPN, but without achieving statistical significance.

**Fig. 2:**
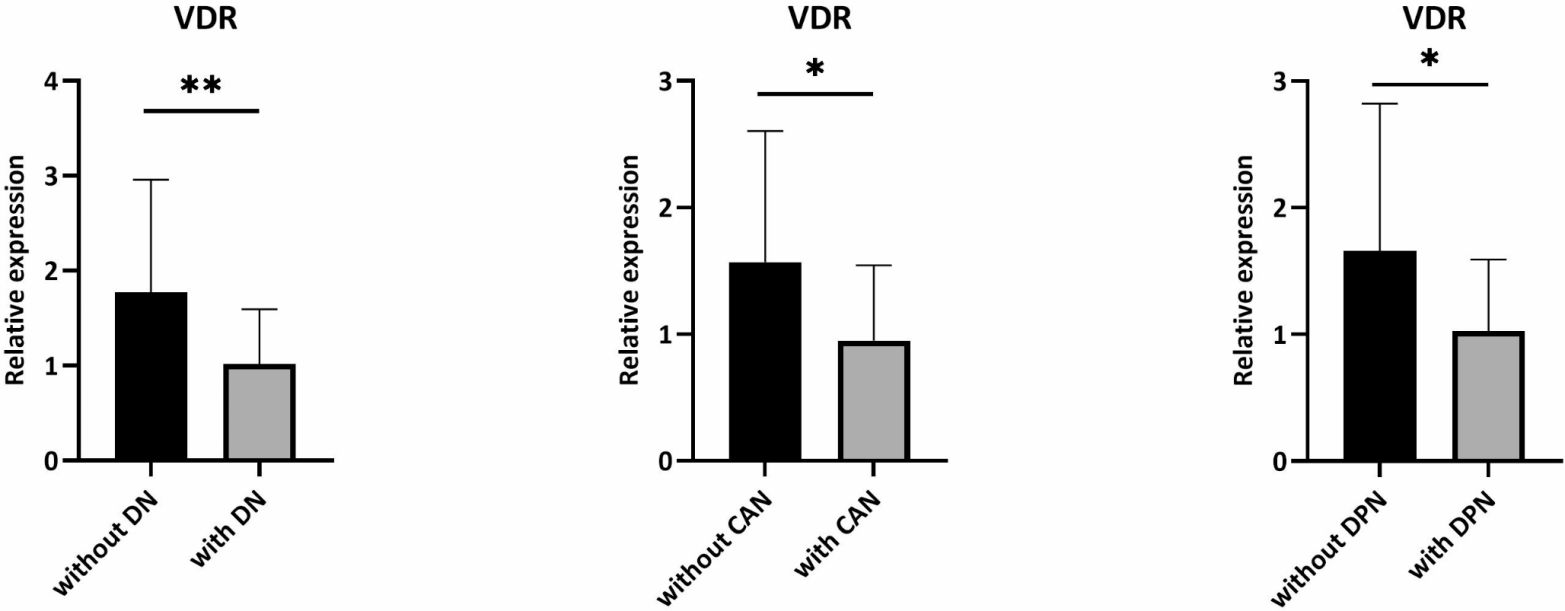
Comparisons of VDR expression levels in different subgroups of diabetic patients. * = p-value < 0.05; **= p-value < 0.01. DN: Diabetic neuropathy; CAN: Cardiovascular autonomic neuropathy; DPN: Diabetic polyneuropathy.

Since VDR seems to play an active role in the regulation of SIRT1 expression, we analyzed VDR expression levels in the same subjects with T2D. Interestingly, the transcript levels of VDR were lower in patients with DN compared to those without DN (*p* = 0.003; p_corr_ = 0.009) (Fig. 2). As shown in Fig. 2, this difference remained significant even when we analysed separately patients with/without CAN (*p* = 0.016) or those with/without DPN (*p* = 0.013). Since we observed a correlation between VDR levels and hypertension (already known in literature [18]), we corrected the analyses also by this variable in a second step, confirming a significant decrease of VDR mRNA in both DPN and CAN groups (p_corr_ = 0.024 and p_corr_ = 0.027, respectively).

Furthermore, we observed a positive correlation between the mRNA levels of SIRT1 and VDR (*p* = 0.01; *r* = 0.384). These results seems to support the hypothesis that VDR might regulate SIRT1 expression.

Then, we evaluated the possible correlation between the expression of these two genes and the neurological scores of T2D patients, both for DPN and CAN. Our analyses showed that the score based on cardiovascular reflex tests (CARTs score) correlated negatively both with SIRT1 (*p* = 0.041; *r* = − 0.309) and VDR (*p* = 0.045; *r* = − 0.282) expression levels. At the light of these correlations, we have performed the same analysis with the single CARTs (Table 2). SIRT1 expression levels resulted also correlated positively with the expiration/inspiration ratio (*p* = 0.016; *r* = 0.362) and with deep breathing (*p* = 0.005; *r* = 0.419), while VDR expression levels correlated negatively with the orthostatic hypotension (*p* = 0.049; *r* = − 0.279). In order to exclude a possible confounding role in this last relationship of drugs potentially interfering in the blood pressure response to standing, as b-blockers, diuretics, nitrates, vasodilators, and a-blockers, we compared the results of the orthostatic hypotension test between the participants with and without these drugs without finding a significant difference. Therefore, it would seem that patients with lower expression levels of these genes have a greater degree of CARTs impairment and more severe CAN.

**Table 2:** Correlation between SIRT1 and VDR expression and neurological parameters.

		CART score	Expiration/Inspiration Ratio	Deep Breathing	Lying to Standing	Valsalva Ratio	Orthostatic Hypotension
SIRT1	Pearson correlation	**− 0.309**	**0.362**	**0.419**	0.062	0.282	−0.272
	p-value	**0.041**	**0.016**	**0.005**	0.694	0.083	0.077
VDR	Pearson correlation	**− 0.282**	0.184	0.234	0.170	0.135	**− 0.279**
	p-value	**0.045**	0.196	0.098	0.237	0.369	**0.049**

Significant correlations are reported in bold

On the contrary, we did not observe associations between the need for supplemental vitamin D and the expression levels of SIRT1 and VDR, nor with the onset of DN.

**Fig. 3:**
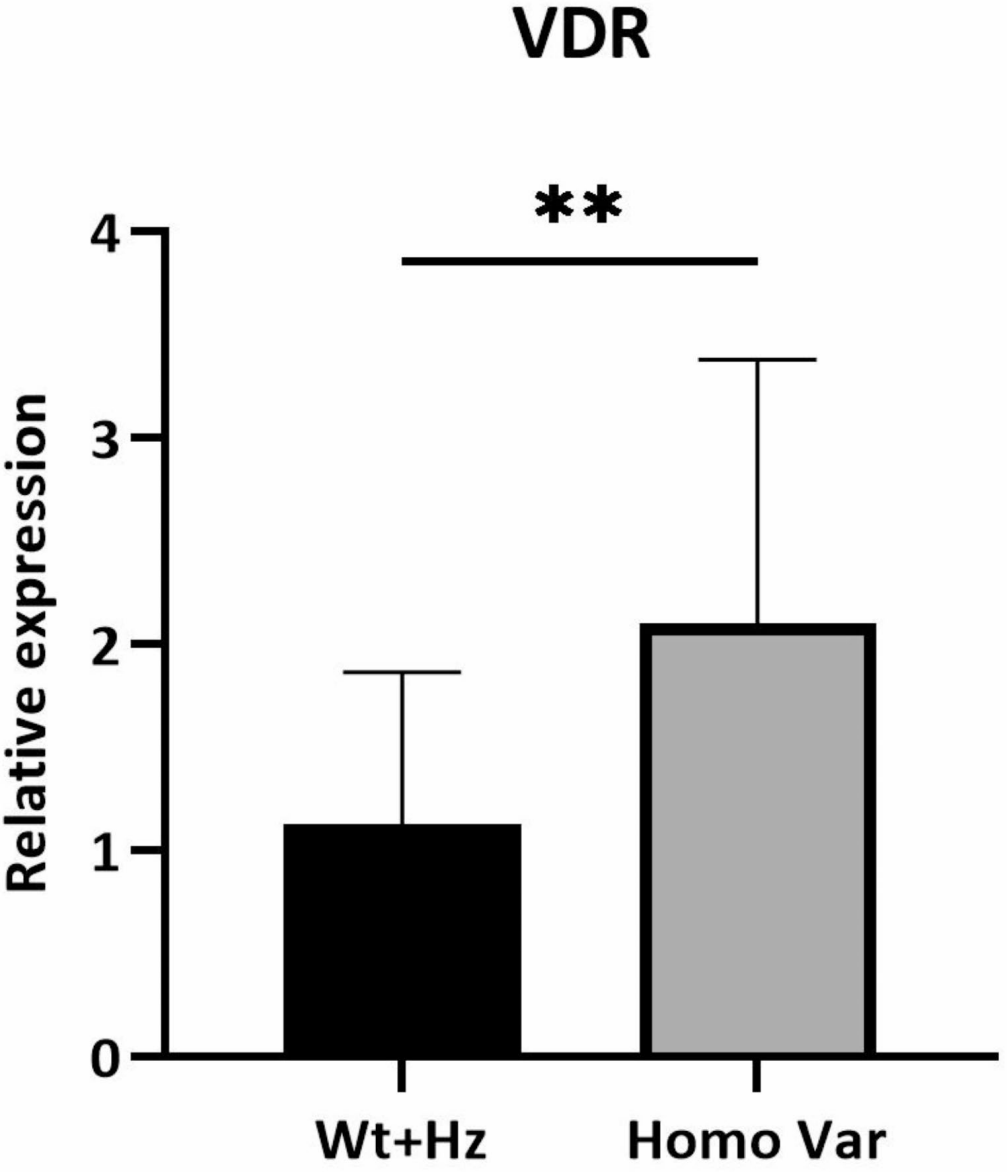
Distribution of mean expression levels of VDR among the genotypic classes for the rs2228570 SNV. **= p-value < 0.01. Wt: Wild-type; HZ: Heterozygous; Homo Var: Homozygous variant.

Lastly, we investigated possible factors that could influence the VDR expression levels. First, we explored the correlation between rs2228570 single nucleotide variant (SNV) localized in the VDR gene promoter region, and the transcripts levels of this gene. We compared the distribution of the mean values of VDR expression in the different genotypes of this SNV, in the whole cohort of analysed subjects (Fig. 3). We found that subjects carrying the TT genotype presented higher mRNA levels of VDR compared to the other genotypic classes (*p* = 0.003).

**Fig. 4:**
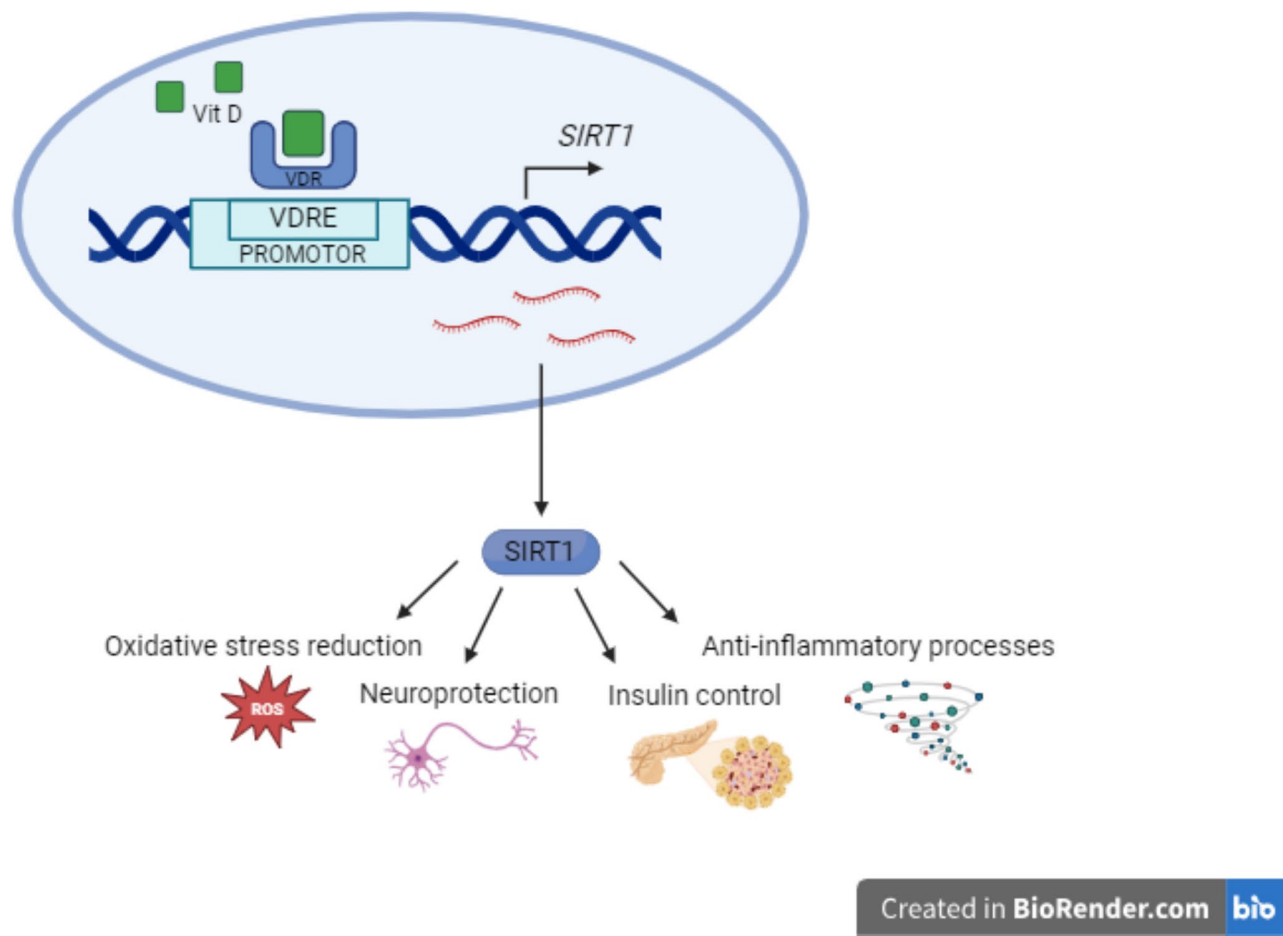
Interplay of VDR and SIRT1. Vitamin D Receptor binds a vitamin D response element (VDRE) in the promoter of the SIRT1 gene and induces its expression.

Secondary, we also evaluated whether the methylation status of VDR promoter region could influence the expression levels, but we found neither correlations between VDR methylation and expression levels, nor a difference in terms of methylation between subjects with and without DN.

## Discussion

Numerous studies have observed that the Sirtuins protein family is involved in the biological processes underlying T2D, such as glucose metabolism, resistance to cellular stress and mitochondrial function [19]. In particular, SIRT1 overexpression seems to improve insulin sensitivity and to reduce insulin resistance [20], while its downregulation inhibits insulin signalling and glucose transport into adipocytes [21]. Furthermore, it has been described that SIRT1 exerts a protective effect on neuroinflammation, and its activation attenuates oxidative stress [22]. These evidences suggested that SIRT1 could play a role in the development of DN and could represent a promising therapeutic target for this condition.

In the present study, we have analyzed the SIRT1 expression levels in peripheral blood of patients with T2D, who were characterized for the presence of DN. We found that T2D subjects with DN, in particular with CAN, have lower levels of SIRT1 expression with respect to those without DN. Our data are consistent with the results described in vitro [11] and in vivo [9] studies, which showed how overexpression of SIRT1 in neurons prevents or reverses DN.

SIRT1 also plays a role in the inflammatory response, inhibiting the secretion of pro-inflammatory molecules: in fact, it has been observed that knockdown of SIRT1 leads to an increase in the secretion of tumor necrosis factor alpha (TNF-α) by macrophages [23]. Accordingly, SIRT1 activation appears to inhibit inflammation by decreasing pro-inflammatory cytokines such as IL-6, NF-κB and ICAM-1 [24]. Interestingly, in vivo studies in obese mice have shown that SIRT1 deficiency increases microvascular inflammation [25], which represents one of the main mechanisms underlying DN.

Furthermore, SIRT1 contributes to cellular tolerance in oxidative stress condition by activating Nuclear erythroid-related factor 2 (Nrf2) to promote the expression of antioxidant genes, such as glutathione S transferase [26], and regulates mitochondrial function through Peroxisome proliferator-activated receptor-gamma coactivator (PGC-1α) [27], with both processes favouring, if altered, the development of DN.

The expression of SIRT1 is controlled by different regulatory mechanisms that can influence its cellular levels. Among these, numerous transcription factors and cofactors have been described, including P53 and hypermethylin cancer 1 (HIC1), which repress SIRT1 transcription, and E2F1, FOXO3a, and C-MYC which promote it [28]. Moreover, several studies showed a direct link between vitamin D and SIRT1 expression, due to binding of VDR to SIRT1 promoter [12]. Indeed, in the promoter of the SIRT1 gene it has been identified a VDRE, to which VDR binds to attract transcription factors and promote gene expression. Our results showed that VDR levels actually decreased in patients with DN, both CAN and DPN. In CAN patients, the expression levels of both genes negatively correlate with a higher disease score, therefore indicating a more severe form of the disease. Furthermore, it is interesting to note that the expression levels of VDR and SIRT1 correlate positively, supporting the direct relationship between the two genes. In fact, in addition to the regulatory mechanism mediated by vitamin D, it has been reported that VDR can induce SIRT1 expression also in a ligand-independent manner, through the interaction with the transcription factors FOXOs [13]. Many of the processes in which VDR is involved, such as reduction of oxidative stress, neuroprotection, anti-inflammatory processes, and insulin control, are shared with SIRT1 and downregulation of both genes could contribute to the onset of neuropathy.

Several studies have described environmental, genetic and epigenetic factors that regulate VDR expression, including methylation status and a functional SNV of the promoter region [29]. Our results confirmed that subjects with rs2228570 TT genotype have higher transcript levels of VDR and it could represent a protective factor against the development of DN.

In this scenario, the decrease in VDR due to predisposing genetic or environmental factors, as well as vitamin D deficiency, could have a negative effect on the transcription of SIRT1 and could influence all the pathways underlying DN regulated by this protein (Fig. 4).

## Conclusion

In conclusion, despite the sample size of our study and the absence of data about vitamin D values in T2D patients constitutes two limitations, our findings highlight the downregulation of SIRT1 and VDR in T2D patients with DN. Although the wide range of biological mechanisms in which SIRT1 and VDR are involved makes it difficult to consider them as potential specific diagnostic and prognostic biomarkers of DN, their observed alteration in the blood of patients with neuropathy certainly provides a new small step in the understanding of this condition. Furthermore, in the context of diabetes, since vitamin D supplementation has been associated with improvements in glycated hemoglobin levels and increased sirtuin1, it is possible to hypothesize a potential role in managing DN by modulating sirtuin levels [30]. Overall, the evidence suggests a significant interaction between vitamin D and sirtuins in various physiological processes, highlighting the potential therapeutic implications of targeting sirtuins through vitamin D supplementation in the management of DN and other associated metabolic disorders.
